# Increased Putamen Volume in Adults with Autism Spectrum Disorder

**DOI:** 10.3389/fnhum.2014.00957

**Published:** 2014-11-25

**Authors:** Wataru Sato, Yasutaka Kubota, Takanori Kochiyama, Shota Uono, Sayaka Yoshimura, Reiko Sawada, Morimitsu Sakihama, Motomi Toichi

**Affiliations:** ^1^The Hakubi Project, Primate Research Institute, Kyoto University, Aichi, Japan; ^2^The Organization for Promoting Developmental Disorder Research, Kyoto, Japan; ^3^Health and Medical Services Center, Shiga University, Shiga, Japan; ^4^Faculty of Human Health Science, Graduate School of Medicine, Kyoto University, Kyoto, Japan; ^5^Rakuwa-kai Otowa Hospital, Kyoto, Japan

**Keywords:** autism spectrum disorder (ASD), MRI volumetry, basal ganglia, caudate, putamen, nucleus accumbens and globus pallidus

## Abstract

Basal ganglia (BG) abnormalities are implicated in the pathophysiology of autism spectrum disorder (ASD). However, studies measuring the volume of the entire BG in individuals with ASD have reported discrepant findings, and no study conducted volume measurement of the entire substructures of the BG (the caudate, putamen, nucleus accumbens, and globus pallidus) in individuals with ASD. We delineated the BG substructures and measured their volumes in 29 adults with ASD without intellectual disabilities and 29 age- and gender-matched typically developed adult controls. We acquired T1-weighted anatomical images and performed semiautomated delineation and volume measurements of the above-mentioned subregions. Total cerebral volumes, sex, and ages were partialed out. Compared with controls, the putamen was significantly larger in the ASD group. The increased volume of the putamen found in high-functioning adults with ASD suggests that structural or histological abnormalities of the putamen may underlie the pathologies of ASD, such as repetitive and stereotyped behaviors and impaired social interactions.

## Introduction

Autism spectrum disorders (ASDs) are heterogeneous neurodevelopmental disorders of unknown etiology. The Diagnostic and Statistical Manual-Fourth Edition-Text Revision (DSM-IV-TR) (American Psychiatric Association, 2000) defines autism or autistic disorder as abnormal behavior in the spheres of communication, social relatedness, and stereotyped behaviors within the first 3 years of life (American Psychiatric Association, 2000). The broader category, pervasive developmental disorder (PDD), includes Asperger’s disorder and a residual category, pervasive developmental disorder not otherwise specified (PDD-NOS). Asperger’s disorder involves pervasive deficits in social interaction and behaviors in the presence of normal verbal development. PDD-NOS is based on social relatedness symptoms but allows for a different age of onset and addresses fewer other spheres. These subcategories are subsumed under the single diagnostic category ASD in the DSM-5 (American Psychiatric Association, 2013).

Repetitive and restrictive behaviors and interests are one of the core clinical manifestations of ASD. It has been postulated that repetitive behaviors/restricted interests are due to abnormalities in the basal ganglia (BG) (Sears et al., 1999; Hikosaka et al., 2002; Hardan et al., 2003; Hollander et al., 2005). The BG is a complex of multiple subcortical regions, including the striatum (composed of three subnuclei: the caudate, putamen, and nucleus accumbens) and the globus pallidus as basic components (Martin, 2003). Abnormalities in the BG have been reported in other neurodevelopmental disorders showing stereotypical motor symptoms, such as obsessive–compulsive disorder (OCD) (Rauch et al., 1997; Kwon et al., 2003; Menzies et al., 2008) and Tourette syndrome (Peterson et al., 2003; Roessner et al., 2011).

The delineation and volume measurement of brain structures using magnetic resonance imaging (MRI) provides valuable information about macroscopic anatomical problems. Previous MRI studies assessing BG volumes in ASD have reported inconsistent findings; some reported no differences in total BG volume (Sears et al., 1999; McAlonan et al., 2002; Hardan et al., 2003), whereas others reported increased volume after controlling for total cerebral volume (TCV) (Herbert et al., 2003; Hollander et al., 2005; Langen et al., 2007). The inconsistent findings suggest that BG abnormalities in ASD are subtle and not apparent by assessing the gross BG volume. Therefore, more detailed analysis of the BG subregions may be needed. To date, no study conducted anatomical delineation and volume measurement of all the aforementioned subregions of the BG. Previous studies have measured the volumes of some of the BG subregions and identified anatomical abnormalities in the specific BG subregions, although inconsistent, possibly depending on the age group or diagnostic subgroups investigated (Hardan et al., 2003; Hollander et al., 2005, Langen et al., 2007, 2009; Qiu et al., 2010). For example, Langen et al. (2007) measured the caudate, putamen, and nucleus accumbens in individuals with ASD and typically developed controls and found the enlarged volumes in the caudate and putamen in the ASD group. A recent study using voxel-based morphometry, which can provide information on microscopic anatomical problems relative to anatomical delineation (Mechelli et al., 2005), also reported that the putamen was larger in adults with ASD (Ecker et al., 2012). Together, these findings suggest that any anatomical abnormality of the BG in adults with ASD might be identified in a specific subregion, such as the putamen.

To investigate this issue, we performed anatomical delineation and volume measurement of the entire BG substructures (the caudate, putamen, nucleus accumbens, and globus pallidus) in adult subjects with ASD without such contaminating factors as intellectual disabilities or language delay, using a semiautomated subcortical segmentation tool for efficient modeling of the brain shape and appearance (Patenaude et al., 2011).

## Materials and Methods

### Subjects

The ASD group comprised 29 adults [5 females, 24 males; age 26.6 ± 8.2 (range 18–46) years] without intellectual disabilities, including 12 (2 females, 10 males) with Asperger’s disorder and 17 (3 females, 14 males) with PDD-NOS who showed milder symptoms than Asperger’s disorder. Therefore, the ASD subjects in this study are considered to have only the core deficits of ASD (i.e., social impairments and repetitive traits). Neurological abnormalities and psychiatric problems other than those associated with ASD (such as clumsiness and circumstantial obsessiveness) were ruled out. The subjects were not taking medication, except one subject on 10 mg paroxetine per day and one on 400 mg sodium valproate per day.

The diagnosis was made using the DSM-IV-TR (American Psychiatric Association, 2000) following a stringent procedure in which every item of the diagnostic criteria was investigated in interviews with the subjects and their parents (and professionals who helped them, if any), conducted by psychiatrists with expertise in developmental disorders (Sayaka Yoshimura, Morimitsu Sakihama, and Motomi Toichi). Only subjects who had at least one of the four social impairment traits (i.e., impairment in non-verbal communication including lack of joint attention, sharing interest, relationships with peers, and emotional and interpersonal mutuality) without satisfying any items of the criteria of autistic disorder, such as language delay, were included. Comprehensive interviews were administered in order to obtain information about the subjects’ developmental histories for diagnostic purposes. Of the total 29 individuals with ASD, 25 individuals completed assessments of the intelligence quotients (IQs) using the Wechsler Adult Intelligence Scale-Revised (WAIS-R). Their levels of symptom severity were also assessed by the psychiatrists (Sayaka Yoshimura, Morimitsu Sakihama, and Motomi Toichi) using the Japanese version of Childhood Autism Rating Scale (CARS) (Schopler et al., 1986), the CARS-Tokyo Version (CARS-TV) with satisfactory reliability and validity (Kurita et al., 1989; Tachimori et al., 2003). Handedness was assessed by the Edinburgh Handedness Inventory (Oldfield, 1971).

The control group comprised 29 adults [5 females, 24 males; age 25.6 ± 4.8 (range 21–43) years]. They were recruited through advertisements and were matched with the ASD group for age and sex. Brief clinical interview with the psychiatrists (Yasutaka Kubota, Sayaka Yoshimura, and Motomi Toichi) ruled out past or present history of neurological, developmental, or psychiatric problems. The IQs and handedness were assessed as in the ASD group.

All subjects had normal or corrected-to-normal visual acuity. After the procedure and purpose of the study were explained fully, all subjects provided informed consent before participating in the study. This study was approved by the local institutional ethics committee.

### MRI acquisition

Image scanning was performed on a 3-T scanning system at the ATR Brain Activity Imaging Center (Magnetom Trio, A Tim System, Siemens) using a 12-channel array coil without acceleration mode. A T1-weighted high-resolution anatomical image was obtained using a magnetization-prepared rapid gradient-echo sequence (TR 2250 ms; TE 3.06 ms; FA 9°; inversion time 1000 ms; FOV 256 × 256 mm; matrix size 256 × 256; voxel size 1 mm × 1 mm × 1 mm; and generalized autocalibrating partially parallel acquisition with an acceleration factor of 2). Elastic pads were placed on each side of the subject’s head to stabilize the head position during image acquisition.

### Image analysis

**Figure 1:**
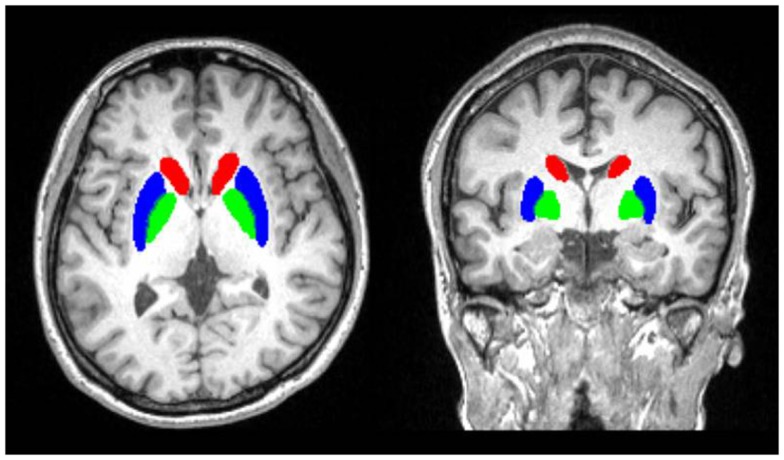
**Illustrations of semiautomated subcortical segmentation**. The area is overlayed on the anatomical MRI of one of the subjects involved in this study.

Segmentation and volumetric analysis of the brain structures was performed with the Integrated Registration and Segmentation Tool (FIRST) in the FMRIB Software Library (FSL) ver. 5 (Patenaude, 2007). FIRST is a semiautomated subcortical segmentation tool used for efficient modeling of the brain shape and appearance within a Bayesian framework (Patenaude et al., 2011). The method has been validated (Patenaude et al., 2011) and has been used in a wide range of applications for volumetric analyses (e.g., Pardini et al., 2014). The analyses were conducted using the standard FIRST procedures (Patenaude et al., 2011). First, the images were registered to a common space based on the Montreal Neurological Institute-152 template using affine registration of the entire head and a subcortical mask to exclude voxels outside the subcortical regions. Then, the inversion transformation was applied to bring the images back into the original space. Finally, the brain structures were segmented based on a Bayesian shape and appearance model, which is part of FSL package and was originally constructed from manually segmented images provided by the Center for Morphometric Analysis, Boston, MA, USA. One of the researchers (Takanori Kochiyama) validated the success of the automated segmentation procedure and inspected the quality of segmentation visually using FSL-provided script to generate summary images in a well-organized webpage format (first_roi_slicesdir). The volumes of the caudate nucleus, putamen, nucleus accumbens, and globus pallidus were extracted for each subject (Figure 1). To control for the TCV, warped modulated gray and white matter images (cf., Ridgway et al., 2011) was obtained using VBM8 software (http://dbm.neuro.uni-jena.de/vbm/) and the sum of gray and white matter volumes was calculated by integrating voxel intensities over the whole segmented image and then multiplying by voxel size. The volumes of each structure were analyzed using a repeated-measures general linear model with group (ASD or control), hemisphere (left or right), sex (male or female), age, and TCV as factors. Main effects or interactions related to the group factor were regarded as effects of interest. Further analyses were conducted using a general linear model (1) without the hemisphere factor and (2) with the group factor including three groups (Asperger’s disorder, PDD-NOS, or controls). Partial correlations holding the TCV effect constant were also calculated between the mean volumes across both hemispheres and between the volumes and CARS scores [calculating the total scores and composite scores for social communication, social interaction, stereotypes and sensory abnormalities, and emotional regulation proposed by Magyar and Pandolfi (2007)]. We conducted exploratory analyses for the shapes of BG substructures (cf., Patenaude et al., 2011) and the volumes of brain regions other than the BG substructures (Patenaude et al., 2011) using the same software. The results were considered statistically significant at *p* < 0.05.

## Results

Background demographics and psychiatric information of the ASD and control groups is summarized in Table 1.

**Table 1:** **Mean (with SD) background information of subjects**.

	ASD						CON	
			
	Total		Asperger		NOS		
*n* total	29		12		17		29	
Female	5		2		3		5	
Right handed	29		12		17		29	
Age	26.6	(8.2)	26.8	(8.8)	26.9	(7.9)	25.6	(4.8)
WAIS-R Full^a^	112.6	(13.9)	113.0	(13.9)	111.8	(14.9)	121.5	(9.0)
Verbal	114.9	(16.0)	117.9	(15.3)	112.5	(17.4)	122.3	(10.6)
Performance	107.4	(13.2)	104.3	(11.7)	109.0	(14.4)	115.6	(11.5)
CARS^a^	22.8	(3.0)	24.0	(3.7)	22.0	(2.3)		

*^a^Investigations were conducted for 25 subjects in the ASD group*.

The CARS scores in the ASD group were comparable to those with high-functioning ASD, including Asperger’s disorder and PDD-NOS, in previous studies (*t*-test, *p* > 0.1) (Koyama et al., 2007; Uono et al., 2011), indicating that the symptoms in the ASD group were sufficiently severe. There was no significant difference in the CARS scores between ASD subgroups (*t*-test, *p* > 0.1). The full-scale IQs of both groups fell within the normal range. All subjects were right handed.

**Figure 2:**
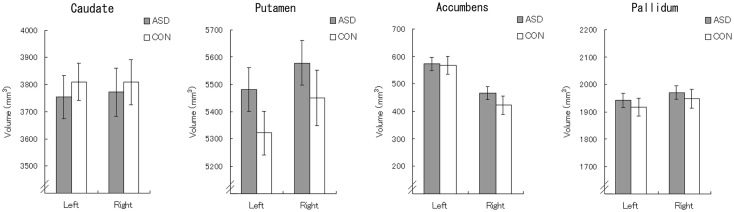
**Mean (±SEM) volumes of the caudate, putamen, globus pallidus, and nucleus accumbens**. *p < 0.05.

For the putamen volumes (Figure 2), a general linear model analysis with group, hemisphere, sex, age, and TCV as factors revealed a significant main effect of group [*F*(1,52) = 4.61, *p* < 0.05], which indicated a higher volume for the ASD group than for the control group. There was no other significant main effect or interaction (*p* > 0.1). To confirm the group difference in the putamen volumes in each hemisphere, we repeated the analysis without the hemisphere factor for the data for each hemisphere, and found that the main effects of group were significant in both hemispheres [*F*(1,52) = 4.10, *p* < 0.05]. For the volumes of the caudate nucleus, nucleus accumbens, and globus pallidus (Figure 2), there were no significant main effects or interactions (*p* > 0.1).

To test the effect of ASD subgroup (Figure S1 in Supplementary Material), a further general linear model was evaluated for the putamen volumes using the same design, except that the group factor included three levels (Asperger’s disorder, PDD-NOS, and controls) and contrasts were conducted across groups. The results confirmed a marginally significant difference between Asperger’s disorder and the controls (*p* < 0.1) and a significant difference between PDD-NOS and the controls (*p* < 0.05), but none between Asperger’s disorder and PDD-NOS (*p* > 0.1).

To test the influence of IQ on putamen volume, partial correlations holding the TCV effect constant were calculated between the mean putamen volumes across both hemispheres and the full-scale/verbal/performance IQs in the ASD group. There were no significant partial correlations between the putamen volumes and IQs (partial *r*s = −0.14, −0.03, and −0.26 for the full-scale, verbal, and performance IQs, respectively; all *p* > 0.1).

To test the relationships between the putamen volumes and psychiatric symptoms, partial correlations (controlling for the effect of TCV) were calculated between the mean putamen volumes across both hemispheres and CARS scores in the ASD group (Table S1 in Supplementary Material). There were no significant partial correlations between the putamen volumes and CARS total score (partial *r* = −0.22, *p* > 0.1) or any of Magyar and Pandolfi’s (2007) composite scores (absolute partial *r* < 0.33, *p* > 0.1).

We conducted some additional exploratory analyses. First, since the software allows analysis of the form of the brain regions, we analyzed the forms of the BG substructures (Figure S2 in Supplementary Material). There were no significant differences between the ASD and control groups in the forms of the caudate nucleus, putamen, nucleus accumbens, and globus pallidus. Then, because the software can output the volumes of other brain structures, including the thalamus, hippocampus, and amygdala of each hemisphere (Table S2 in Supplementary Material), we analyzed the volumes of these regions using the same methods. There were no significant main effects or interactions related to the group factor for any regions (all *p* > 0.1).

## Discussion

To our knowledge, this is the first delineation study on the whole basic BG substructures (the caudate nucleus, putamen, nucleus accumbens, and globus pallidus) that identified increased putamen volume in well-characterized adults with ASD. Individuals with ASD have problems with social interaction and non-verbal communication as children and adults. Although follow-up studies report improved adaptive functioning in adulthood (Magiati et al., 2014), many adults with ASD have considerable psychosocial impairment (Hofvander et al., 2009; Mordre et al., 2012). Our findings are important for elucidating the neuroanatomical basis of the residual autistic traits in adults in this population.

The present results of increased putamen volumes in ASD are consistent with the findings in several previous anatomical delineation studies (e.g., Langen et al., 2007) and voxel-based morphometry studies (e.g., Ecker et al., 2012). However, other anatomical delineation studies (e.g., Hollander et al., 2005; Qiu et al., 2010) and voxel-based morphometry studies (e.g., Nickl-Jockschat et al., 2012; Greimel et al., 2013) reported different results. These inconsistent findings may be partly attributable to some methodological differences, such as differences in ages, diagnostic subcategories, and data analyses. Specifically, Qiu et al. (2010) tested 32 boys aged 8–12 with ASD and reported a non-significant trend toward a putamen volume decrease. These discrepancies may be attributable to the difference in the developmental stage. Typically developing male children show an increase in the putamen with age, even after adjusting for TCV (Giedd et al., 1996). A recent longitudinal study observed that the growth rate of the striatum was faster in 9- to 12-year-old children with ASD compared to typically developing children, and faster striatal growth was correlated with more severe repetitive behavior at preschool ages (Langen et al., 2014). Since the ASD subjects of the current study were adults, accelerated growth of the putamen volumes may be more pronounced than those of previous studies. Hollander et al. (2005) reported volume increases in the caudate and a near-threshold trend of the putamen volume increases in 17 adults with ASD. These results may be accounted for by differences in subgroups. While Hollander et al. (2005) included nine with autistic disorder, seven with Asperger’s disorder, and one with PDD-NOS, our subjects included 12 subjects with Asperger’s disorder and 17 subjects with PDD-NOS. The caudate volume increases were reported mostly in studies on individuals with autistic disorders [Sears et al., 1999; Rojas et al., 2006; Langen et al., 2014; for review, see Stanfield et al. (2008)]. These findings might reflect more distinct autistic traits in these populations, such as motor stereotypes as well as abnormalities in verbal/communicative domain. Greimel et al. (2013) conducted whole-brain voxel-based morphometry analysis on 26 subjects with Asperger syndrome, 20 with high-functioning autism, and one with atypical autism according to ICD-10 (World Health Organization, 1993), but did find enlarged volumes in the putamen compared to controls. The discrepancy may be explained from the fact that the whole-brain voxel-based morphometry approach is less sensitive in finding differences in an *a priori* specified region in the ROI-based approach. Given anatomical abnormalities in ASD distributed in a structure such as the putamen, there may be no single voxel in a general linear model analysis that survives statistical threshold. Further studies using other suitable methodology for analyzing subcortical structures, such as machine learning-based automated segmentation (Powell et al., 2008) are warranted.

Our correlation data using CARS measures did not show clear evidence about the functional significance of this anatomical problem in the ASD group. The results could be accounted for the limitation that CARS lack the items for a repetitive and stereotyped pattern of behaviors (Schopler et al., 1986). Regarding this issue, a previous study reported that total putamen volumes were positively correlated with repetitive behavior scores in ASD (Hollander et al., 2005). Anatomical abnormalities of the putamen were also reported in a variety of psychiatric/neurodevelopmental disorders showing repetitive/obsessive symptoms, including OCD, Tourette syndrome, and schizophrenic disorders (Chemerinski et al., 2013). For example, increased putamen volumes were repeatedly observed in subjects with OCD [for review, see Rotge et al. (2010)]. Together with these findings, we speculate that BG pathology confined to the putamen may be related to the repetitive behavior/restricted interest in our subjects, but as yet it is not possible to define the direction of this association. Further studies are needed in order to investigate possible relationship between the observed abnormality in the putamen and the clinical phenotype of repetitiveness in adult individuals with ASD.

The human putamen receives associative, motor, and limbic projections (Haber, 2003; Bernácer et al., 2012) and performs integrative information processing underlying various tasks, such as motor control (Lehéricy et al., 2006) and cognitive and emotional processing (Monchi et al., 2001; Haber et al., 2006). Interestingly, functional neuroimaging studies of ASD demonstrate abnormal activity of the striate (the lateral parts correspond to the putamen and caudate) during social processing (Dapretto et al., 2006; Masten et al., 2011; Weng et al., 2011; Delmonte et al., 2012) and reward processing tasks (Scott-Van Zeeland et al., 2010; Delmonte et al., 2012). Furthermore, diffusion tensor imaging studies have shown decreased white matter connectivity between the striatum and prefrontal cortex in ASD compared with typically developed individuals (Langen et al., 2012). These data suggest that the putamen abnormality observed in ASD might be related not only to the domain of motor/repetitive behaviors *per se* but also to broader ASD phenotypes, such as impairments in higher cognitive functioning, including emotional facial recognition and reciprocal social interaction.

Several limitations of this study need to be mentioned. First, it included a modest number of high-functioning adults with Asperger’s disorder or PDD-NOS, and the results should be interpreted with caution. Since our sample did not include subjects with autistic disorder, it is not clear whether our findings can be extended to low-functioning adults with autistic disorder. Second, the use of a cross-sectional design limits the developmental conclusions that can be drawn from the present findings. Further longitudinal studies are needed to explore the specific neurodevelopmental pattern of putamen growth in ASD. Finally, the volumetric approach is clearly limited in clarifying the functional abnormality of the BG that is involved in broader cortico–subcortical networks. Further neuroimaging and electrophysiological studies targeting possible relationship between neural circuitries involving the putamen and higher cognitive processing in ASD, especially in the domain of social interaction and/or decision making, are needed.

## Conflict of Interest Statement

The authors declare that the research was conducted in the absence of any commercial or financial relationships that could be construed as a potential conflict of interest.

## Supplementary Material

The Supplementary Material for this article can be found online at http://www.frontiersin.org/Journal/10.3389/fnhum.2014.00957/abstract
