# Supplementary figures and images for: Increased Putamen Volume in Adults with Autism Spectrum Disorder
Source: Front Hum Neurosci. 2014 Nov 25;8:957. doi: 10.3389/fnhum.2014.00957 (PMC4243557; doi:10.3389/fnhum.2014.00957)

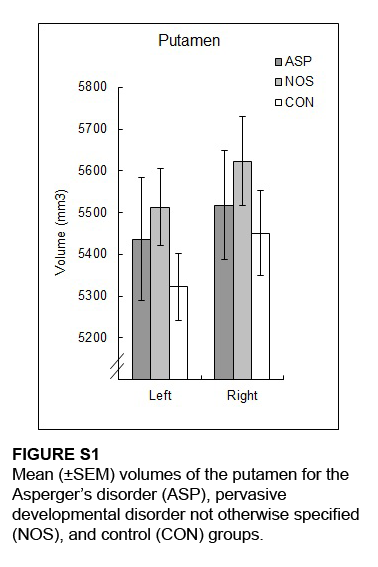

Supplement: Supplementary file 3 [file Image1.TIF]

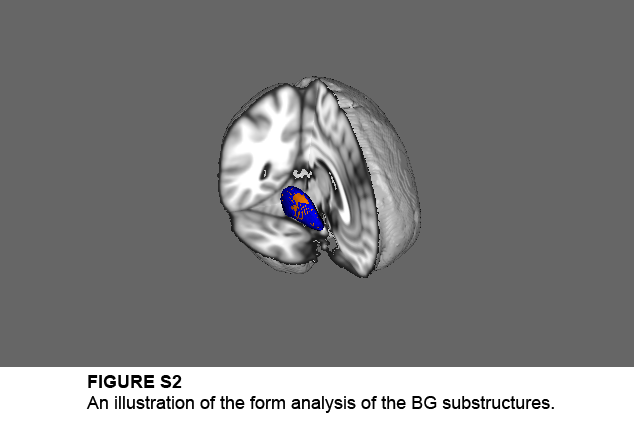

Supplement: Supplementary file 4 [file Image2.TIF]
